# Identifying, studying and making good use of macromolecular crystals

**DOI:** 10.1107/S2053230X14016574

**Published:** 2014-07-25

**Authors:** Guillermo Calero, Aina E. Cohen, Joseph R. Luft, Janet Newman, Edward H. Snell

**Affiliations:** a Department of Structural Biology, University of Pittsburgh Medical School, Pittsburgh, PA 15261, USA; b Stanford Synchrotron Radiation Lightsource, SLAC National Accelerator Laboratory, Stanford University, Menlo Park, CA 94025, USA; c Hauptman–Woodward Medical Research Institute, 700 Ellicott Street, Buffalo, NY 14203, USA; d Department of Structural Biology, State University of New York at Buffalo, 700 Ellicott Street, Buffalo, NY 14203, USA; e CSIRO Collaborative Crystallisation Centre, 343 Royal Parade, Parkville, Victoria 3052, Australia

**Keywords:** crystal detection, crystal growth, crystal positioning

## Abstract

Structural biology has contributed tremendous knowledge to the understanding of life on the molecular scale. The Protein Data Bank, a depository of this structural knowledge, currently contains over 100 000 protein structures, with the majority stemming from X-ray crystallography. As the name might suggest, crystallography requires crystals. As detectors become more sensitive and X-ray sources more intense, the notion of a crystal is gradually changing from one large enough to embellish expensive jewellery to objects that have external dimensions of the order of the wavelength of visible light. Identifying these crystals is a prerequisite to their study. This paper discusses developments in identifying these crystals during crystallization screening and distinguishing them from other potential outcomes. The practical aspects of ensuring that once a crystal is identified it can then be positioned in the X-ray beam for data collection are also addressed.

## Abstract 2

As technology advances, the crystal volume that can be used to collect useful X-ray diffraction data decreases. The technologies available to detect and study growing crystals beyond the optical resolution limit and methods to successfully place the crystal into the X-ray beam are discussed.

## 1. Introduction   

The relationship between the intensity of the X-ray data produced and the volume of the crystal was originally captured by Darwin’s formula (Darwin, 1922 ▶),
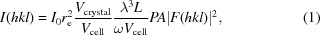
where *I*(*hkl*) is the intensity of a fully recorded reflection, *I*_0_ is the intensity of the incident beam, *r*_e_ is the classical electron radius, *V*_crystal_ is the illuminated crystal volume, *V*_cell_ is the unit-cell volume, λ is the X-ray wavelength, *L* is the Lorentz factor, ω is the rotation speed, *P* is a polarization factor, *A* is an absorption factor and *F*(*hkl*) is the structure amplitude. This assumes a single-crystal data-collection methodology where the crystal is rotated in a monochromatic X-ray beam. The reflection intensity scales as a function of the incident intensity and decreases as a function of the illuminated volume, with a doubling in intensity allowing similar data to be obtained from a crystal of half the volume, radiation-damage considerations aside. From these considerations alone it is immediately apparent that going from an incident brilliance of approximately 10^8^–10^9^ photons s^−1^ mrad^−2^ (0.1% bandwidth)^−1^ for a laboratory system to values approaching 10^20^ for a third-generation undulator source considerably decreases the volume of the crystal required to provide useable *I*(*hkl*)s. If we consider X-ray free-electron laser sources (XFELs) with a brilliance of ∼10^30^, the volume required decreases still further to the point where nanocrystals are used and delivered to the beam using a liquid jet.

In a recent comprehensive article, Giegé (2013 ▶) describes the first crystallization experiments where crystals were visible to the naked eye and had edge dimensions of millimetres. Sealed-tube X-ray sources enabled the study of these millimetre-scale crystals; when more advanced rotating-anode sources, coupled with microfocus optics, were developed,‘ this reduced the X-ray beam diameters (and thus the required crystal size) to a few hundred micrometres. The second generation of synchrotrons, dedicated as experimental light sources, produced similar size beams. The introduction of third-generation synchrotrons, utilizing insertion devices, has reduced useable beam diameters while increasing flux, thus further reducing the required crystalline volume. Using a focused 30 µm X-ray beam (Cusack *et al.*, 1998 ▶), X-ray data to 2.5 Å resolution were collected from hexagonal crystals of bacteriorhodopsin with dimensions ∼30 × 30 × 5 µm (Pebay-Peyroula *et al.*, 1997 ▶); more recently, using a 20 µm X-ray beam at SSRL, structural information was obtained from semi-synthetic ribonuclease S with crystals of dimensions ∼20 × 5 × 1 µm (Fafarman & Boxer, 2010 ▶).

X-ray free-electron lasers have taken this limit further in the study of nanocrystals of photosystem I, where a crystal suspension was injected into a 70 fs duration 30 Hz X-ray Linear Coherent Light Source (LCLS) beam (Chapman *et al.*, 2011 ▶). Structural data were collected from crystals as small as 250 nm (estimated from the fringes between the diffraction peaks). This is even more remarkable when considering the unit-cell parameters of these crystals, *a* = *b* = 281, *c* = 162 Å, and suggests that even smaller crystals will have the potential to provide equal, or better quality, data in the future. Historically, in just over 50 years, we have moved from X-ray data collection from macromolecular crystals measured in millimetres to those measured in nanometres.

Until recently, visible-light microscopy was adequate to identify and characterize crystals that were suitable for X-ray diffraction. Conventional visible microscopy has a resolution given by
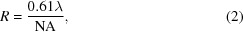
where NA is the numerical aperture and λ is the wavelength of the incident light. This makes the assumption that the condenser and objective lenses have the same NA, which is typically a maximum of ∼0.95 in air (Lipson *et al.*, 1995 ▶). Using this, the effective maximum resolution of an optical microscope, *i.e.* the ability to distinguish two objects, is ∼250 nm at the violet end of the visible spectrum; in practice, the minimum size object that can be resolved is much larger than this, on the order of micrometres. However, even if we could observe crystals at the theoretical limit, we can now collect useful diffraction data from crystals smaller than these. This presents clear problems when working with nanocrystals (those whose dimensions are measured in nanometres): how do you tell when you have them, how do you study their growth and, once you have them, how do you accurately position them in an X-ray beam?

While there are theoretical limits to the volume of a crystal that can produce X-ray data (Holton & Frankel, 2010 ▶), there is already a need to think beyond visible microscopy methods to follow the crystallization process, to identify crystals, and to make practical use of these crystals given the capabilities of existing and future X-ray sources. In this paper, we discuss what visible microscopy tells us about crystallization and describe other optical techniques that supplement or extend this information. We then describe complementary approaches that enable the detailed study of nanocrystallization. Finally, we show how approaches used to study crystallization can be combined with other methods to facilitate the study of crystals with ever-decreasing volumes.

## 2. Crystallization   

### 2.1. Optical microscopy in crystallization screening   

#### 2.1.1. Observation of outcome   

**Figure 1:**
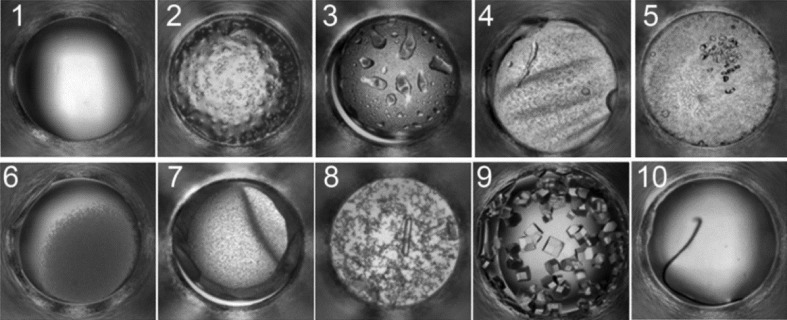
A representative example of typical crystallization screening outcomes; a description of these outcomes is given in the text. Reprinted with permission from Luft, Wolfley *et al.* (2011 ▶). Copyright (2011) American Chemical Society. Each well has a diameter of 0.9 mm.

Up to the turn of the century, observation of the crystallization process was invariably *via* an optical microscope, and the results of the experiments would be recorded as text notes, with perhaps the addition of the occasional microphotograph. Many research groups now use automatic digital imaging, and the interpretation of the experimental outcome is performed indirectly on the image rather than directly from the experiment. Whether observed directly or from images, the information determined from the experiments is similar: the number, size and morphology of any crystals that may form. However, crystals are the exception; a range of outcomes may occur which represent the phase diagram of the macromolecule under the diverse biochemical conditions of crystallization screening. As an example, in Fig. 1 ▶ outcomes are shown for crystallization screening experiments conducted at the Hauptman–Woodward Medical Research Institute’s High-Throughput Crystallization Screening (HTS) laboratory (Luft *et al.*, 2003 ▶; Luft, Snell *et al.*, 2011 ▶). These examples illustrate the most common outcomes for a group of 147 456 (96 proteins × 1536 cocktails) crystallization experiments. The ten most common outcomes are shown: type 1 is clear; type 2 is phase separation; type 3 is phase separation and precipitate; type 4 is phase separation and skin; type 5 is phase separation and crystals; type 6 is precipitate; type 7 is precipitate and skin; type 8 is precipitate and crystal; type 9 is crystals; and type 10 is anything that is undefined or possible contamination (Snell, Luft *et al.*, 2008 ▶; Snell, Lauricella *et al.*, 2008 ▶). These outcomes and their relative locations on the protein solubility map have been described in detail elsewhere (Luft, Wolfley *et al.*, 2011 ▶). With a coarse sampling of biochemical and/or biophysical space, a condition that produced a result that is identified as a crystal, or shows potential to lead to a crystal, would be expanded upon by variation of the original experimental conditions; this process is known as optimization. Where there is a higher density of sampling of the biochemical space and/or biophysical space, the surrounding results may point to an appropriate experimental vector for this process, minimizing the subsequent experimental sampling (Snell, Nagel *et al.*, 2008 ▶). In the images in Fig. 1 ▶ the drop volume (protein and precipitate) is 400 nl and the diameter of the drop is 0.9 mm. Crystals with dimensions of the order of several micrometres can be imaged, but not necessarily resolved, such as is the case for granular precipitation or crystalline outcomes obscured by the presence of, for example, dense precipitate.

**Figure 2:**
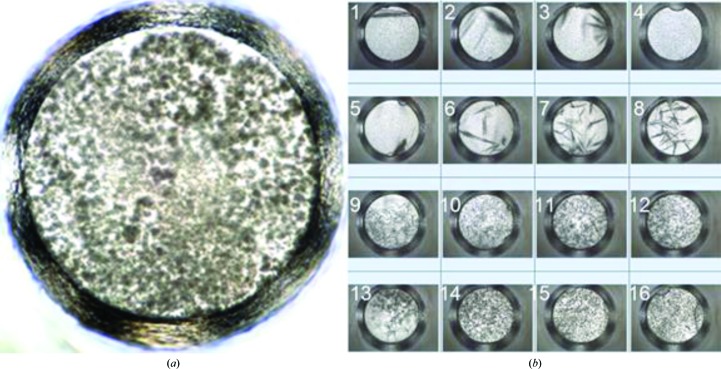
Example of an initial screening hit: (*a*) a patterned precipitate that owing to the poor resolution may at first glance not seem worthy of pursuit and (*b*) a progression of 16 experiments with decreasing protein concentration and increasing precipitant concentration produced by manipulating the ratio of protein volume to precipitate volume used in the original experimental drop (*a*). As the protein volume decreases and the precipitant volume increases, a point is reached where single crystals can clearly be observed from the visible images. The optimization process reveals that the original precipitate was microcrystalline. As in Fig. 1 ▶, each well has a diameter of 0.9 mm.

The characterization of outcomes and the optimization strategies for crystal lead conditions depends on the quality of the imaging system. A low-powered microscope may not provide sufficient magnification to allow the identification of a granular precipitate, for example. This can have a significant impact. Fig. 2 ▶ shows a series of images where one of the outcomes initially produced in the screen is a precipitate that initially may not appear to be interesting (Fig. 2 ▶ *a*). By varying the protein and precipitant concentration (Fig. 2*b*), a visually high-quality crystal can be produced, showing that the initial hit was indeed useful. This method of validation requires extra experimental steps and protein material, and it is generally not practical to pursue this approach for all precipitates observed from even a small (96-cocktail) screening experiment. This said, if the promising precipitated outcomes (*e.g.* microcrystalline precipitate) could be easily identified, then this approach of systematically varying the ratio of the protein and precipitant is likely to be successful in a significant number of cases. If not, further suggestions are provided in the companion paper (Luft *et al.*, 2014 ▶). Ideally, we would like to distinguish microcrystalline precipitation using image data alone.

Typically, visible microscopy or digital images are interpreted by human observation, despite a significant body of work to develop automated image-analysis systems to annotate the digital images. The fewer annotation options, the more likely an automated system (or a human) is to be consistent. How many outcomes need to be distinguished to be useful? ‘Crystal’ or ‘not crystal’ would appear to be the minimum requirement and can be a successful approach in many cases, but even this is nontrivial. Outcomes other than ‘crystal’ can guide optimization. For example, consider clear drops. While these can be simply classified by observation, from this alone it is not possible to identify the location of the outcome on a phase diagram: a clear drop could mean several things from a thermodynamic perspective. The drop could be undersaturated, it could be saturated, or it could be insufficiently supersaturated (metastable) to undergo a spontaneous, homogeneous nucleation event in the absence of any heterogeneous nucleant. How can we discern the difference when visually all of the drops appear identical? We can look at the drops in the context of their chemical relationships to other experiments. If a clear drop is biochemically adjacent to a precipitated drop, then it is more likely to be near the labile zone where spontaneous nucleation is thermodynamically and kinetically feasible. We can test this hypothesis using seeding protocols to initiate crystal growth. We can also slightly increase the level of supersaturation by changing the temperature, increasing the concentration of precipitants or altering the pH. Clear drops which are adjacent to precipitated drops (in a grid of precipitant) can be chemically very close to conditions that will produce crystals. Similarly, precipitates can be further investigated; for example, by adding water to see if they dissolve, which can be indicative of a ‘good/microcrystalline precipitate’ with the protein properly folded. If the precipitate absorbs a dye, such as methylene blue, it is likely to be microcrystalline rather than amorphous precipitate. Another consideration is the homogeneity of the precipitation pattern. It is often the case that a patterned precipitate, as shown in Fig. 2 ▶(*a*), is indicative of a microcrystalline condition. The analysis of results by visual imaging requires the comparison of outcomes, knowledge of chemistry, and knowledge of how that chemistry may influence the protein solubility.

Visible microscopy has an advantage in that it is (or should be) a common instrument in every crystallization laboratory. This means that it can be used repeatedly to follow the course of crystallization experiments and to identify changes over time either manually or, with more sophisticated imaging systems, automatically (Mele *et al.*, 2014 ▶). Given enough sample, time, careful experimental design, and consistent observation, a phase diagram of a protein can be constructed, allowing rational crystallization from microscopic studies alone.

#### 2.1.2. Use of polarization   

**Figure 3:**
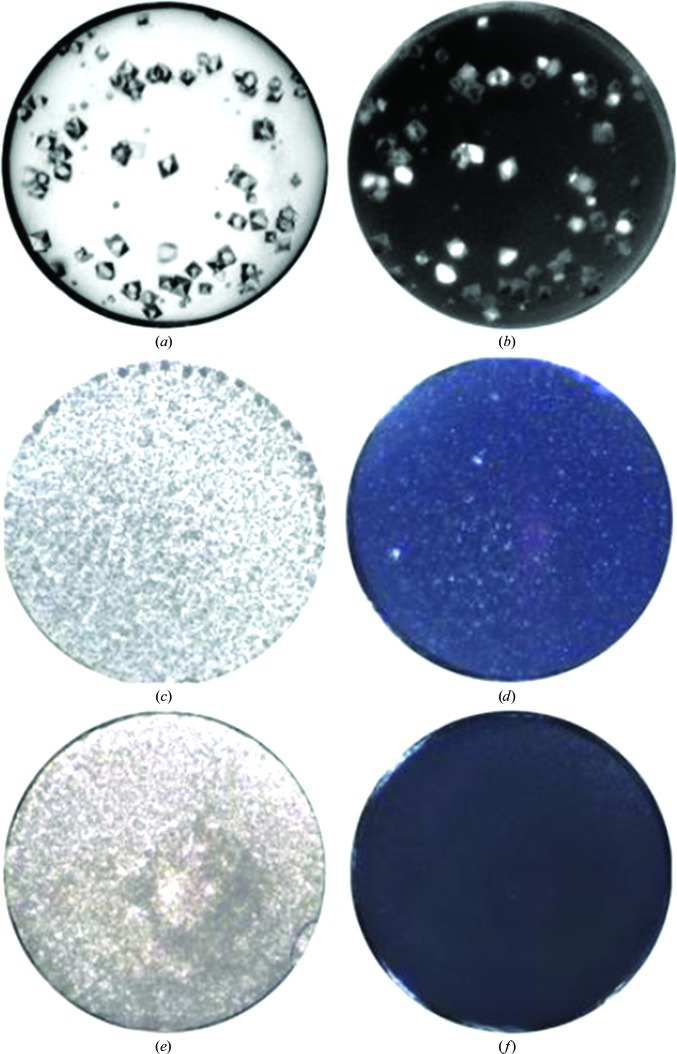
An example of the use of a polarized image. The images on the left were taken with standard imaging. (*a*) shows an experiment containing clearly visible crystals and (*b*) their view under crossed polarizers. (*c*) shows a drop containing microcrystalline precipitate, while (*d*) shows the same drop imaged with crossed polarizers. Notice the soft glow of the precipitate and bright objects that denote larger crystals. (*e*) shows an amorphous precipitate; note that the corresponding cross-polarized image (*f*) does not show any evidence of birefringence. Each well has a diameter of 0.9 mm.

For many crystals, their refractive index depends on the polarization of the light illuminating them: this property is called birefringence. If a light microscope is set up with a polarizer and an analyzer in the light path, then crystalline precipitate may be identified through birefringence, which is seen as a soft glow as the analyzer is rotated (Fig. 3 ▶). Unfortunately, birefringence is a property of both salt and macromolecular crystals and is not seen in cubic crystal systems. Using birefringence in practice can be stymied by the optical properties of the plastic plates used in crystallization that may mask any birefringence from the crystals. The disadvantages of missing a small number of cases, or a false positive, is outweighed by the advantages of discovering a crystalline object; once a good microscope is available, the addition of a polarizer and analyser is a fraction of the initial investment.

#### 2.1.3. Importance of color   

**Figure 4:**
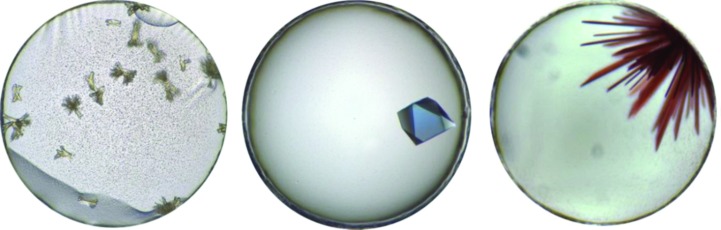
Color crystal images. These images are examples of three different proteins each having color, in this case yellow, blue or red, caused by the oxidation state of a bound ligand or metal ion. Using black-and-white images, it is often impossible to distinguish a particular oxidation state which could be critical for the interpretation of functional studies from crystallographic data. Each well has a diameter of 0.9 mm.

Many digital imaging systems record in black and white to maximize resolution and minimize file size, and the widespread use of monochrome images has downplayed the importance of color in observing crystallization-trial results. The number of colored proteins is small, but color can indicate the presence of metal ions or other ligands bound to the protein. Color can also be engineered into the sample with a ‘crystallization tag’ (Suzuki *et al.*, 2010 ▶). It can also, through the oxidation state of the metal, inform the crystallographer on the functional state of the protein under particular chemical conditions. In the case of a colored protein, the color can sometimes help to distinguish microcrystalline precipitates from denatured precipitates, with the former having the appropriate color and the latter often appearing brown (Fig. 4 ▶). If color imaging is not available digitally, it is highly recommended to view results that may be of interest through a microscope

#### 2.1.4. Use of other wavelengths   

**Figure 5:**
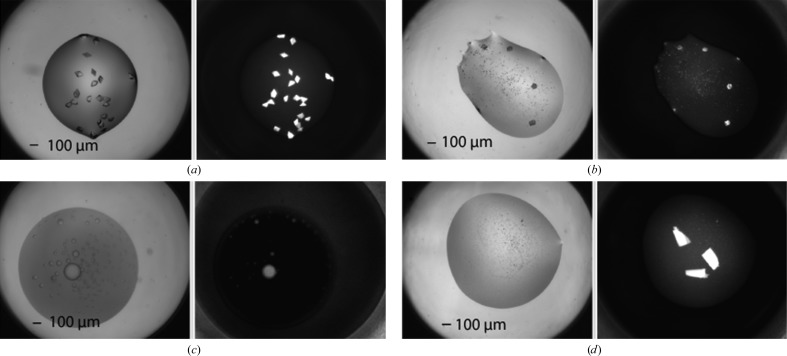
Examples of UV-imaging outcomes with the associated bright-field (visible) image: (*a*) the good, protein crystals fluorescing strongly (proteinase K) also easily identified visually, (*b*) the bad, salt crystals (calcium sulfate) with adsorbed protein, (*c*) the ugly, noncrystal information showing phase separation (also a potential lead condition for optimization) in a myoglobin-containing trial (myoglobin is excluded from one phase) and (*d*) the beautiful, an example of the identification of protein crystals that may have been easily missed visually. In each case a 100 µm scale bar is visible in the bottom left corner.

**Figure 6:**
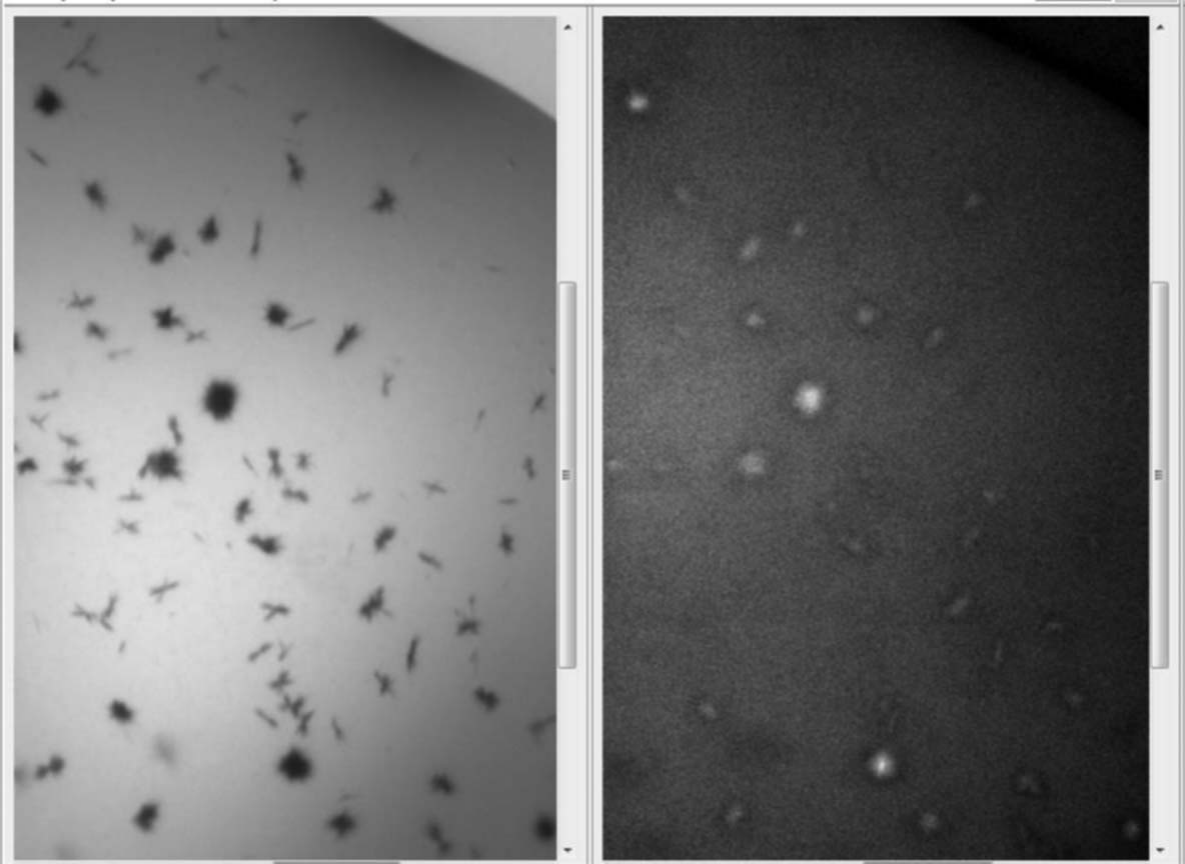
Despeckling (median filtering) applied by default to UV images can remove the signal from small protein crystals. During the process of median filtering, a pixel’s value is replaced by the median value of the pixels around it. This image was taken at a magnification such that one pixel is approximately 1 µm, so that the very thin needles are approximately 1–2 µm wide and thus are removed during the median filtering step.

Infrared imaging was first used to study cryocooling techniques (Snell *et al.*, 2002 ▶) and later to identify crystals in a vitrified loop (Snell *et al.*, 2005 ▶), but the process required expensive instrumentation. At about the same time, intrinsic ultraviolet fluorescence was developed to identify and distinguish protein crystals (Judge *et al.*, 2005 ▶) and it was later used to investigate systems in hydrated bilayers (Lunde *et al.*, 2005 ▶). The amino acid tryptophan contained in many proteins absorbs ultraviolet light in the range 260–320 nm (mostly 280–290 nm) and emits light in the region 300–450 nm, with peak emission at 340–360 nm (Lakowicz, 1999 ▶; Permyakov, 1993 ▶). By illuminating crystallization experiments with an ultraviolet-light source and detecting the fluorescent signal, crystals of proteins containing tryptophan can be identified even in the presence of precipitate and distinguished from (nonfluorescing) salt crystals. The technique assumes that the local concentration of protein is greatest in a crystal and thus crystals should produce more signal than the background. The results have been quite successful in distinguishing protein from salt, characterizing phase separation and identifying protein crystals in precipitate (Fig. 5 ▶). A detailed study of UV imaging by the Collaborative Crystallisation Centre (C3) has resulted in a set of practical guidelines (Desbois *et al.*, 2013 ▶). It is necessary that the proteins being imaged contain tryptophan, but not sufficient: the signal can be quenched by other structural features, for example the presence of a haem group in the protein or of some metal centers. Tryptophan fluorescence is highly sensitive to the local environment of the tryptophan side chain, so that proteins of similar size and containing the same number of tryptophans may respond quite differently to UV light. Salt crystals may emulate fluorescing protein crystals if protein adheres to the surface (Stevenson *et al.*, 2014 ▶), but this can sometimes be distinguished from the uniform glow of a protein crystal. The crystallization cocktail itself can influence the success of the technique; *e.g.* the presence of nitrate ions completely quenches the fluorescence from tryptophan. Another problem that UV imaging faces is background noise from dust, which can fluoresce brightly. Noise in the UV images may be reduced by a despeckling (median filtering) step. While this will reduce noise from interference, it will also remove the signal from very tiny crystals (Fig. 6 ▶).

Tryptophan makes up an average of 1.09% of the residues in proteins (Gilis *et al.*, 2001 ▶), but there are macromolecules that do not contain this amino acid. Fluorescent dyes can be added by covalently modifying the macromolecule (Forsythe *et al.*, 2006 ▶) or by the addition of nonspecific dyes during the crystallization process, allowing the detection of crystals as small as 1 µm in one dimension (Groves *et al.*, 2007 ▶; Watts *et al.*, 2010 ▶). UV or other wavelength light provides an orthogonal observation to a visible-light observation, increasing the confidence with which a result can be associated with a particular trial.

#### 2.1.5. Other optical techniques   

*Second-order nonlinear optical imaging of chiral crystals*. While visible observations make use of the linear properties of light, nonlinear optical processes have also been used to detect crystals in the presence of precipitate or to identify crystals that were too small for visual observation. Most chiral crystals generate a weak second harmonic (frequency doubling) of incident light. This effect has been exploited in a technology dubbed second-order nonlinear optical imaging of chiral crystals (SONICC; Wampler *et al.*, 2008 ▶; Kissick *et al.*, 2011 ▶), where an intense femtosecond pulse from a laser source illuminates a crystallization trial and a detector sensitive to the doubled frequency is used to detect the signal. This can be a very sensitive technique; current instruments are estimated to be able to detect crystals as small as 90 nm (0.09 µm) in all dimensions, with the potential to detect even smaller samples (Kissick *et al.*, 2011 ▶). A small number of biological crystals are nonchiral and a larger number of nonprotein crystals are chiral (*e.g.* detergents and drug-like small molecules), but other sensitive methods are available to discriminate between a biological or small-molecule crystal (Closser *et al.*, 2013 ▶).

SONICC has proved to be especially powerful when imaging crystals grown in lipidic mesophases, for example membrane proteins. Crystals from mesophases tend to be small, and if colorless are often masked by the interaction of lipidic cubic phase with the crystallization cocktail. A trial of the SONICC technique on samples crystallized in lipidic phases, coupled with automated scoring compared with manual inspection of bright-field and birefringent images, demonstrated that SONICC was more sensitive and was able to identify larger crystals (∼15 µm in size), small crystals (between 2 and 15 µm) and showers of crystals (less than 2 µm and spaced less than 2 µm apart). Out of 41 crystallization experiments studied, SONICC performed better than the more conventional imaging ∼70% of the time and identified crystalline material in nine experiments where bright-field and birefringent inspection failed to reveal positive results (Kissick *et al.*, 2010 ▶).

*Two-photon fluorescence*. An extension to SONICC has been the addition of two-photon fluorescence, which is used to help understand the crystallization process by studying the distribution of impurities within protein crystals (Caylor *et al.*, 1999 ▶). In this case the illuminating light is visible (515 nm) and the fluorescent light is shifted to the near-UV (∼340–380 nm; Madden *et al.*, 2011 ▶). Two-photon fluorescence has been shown to identify SONICC-silent crystals (Padayatti *et al.*, 2012 ▶). Because two-photon fluorescence uses a beam-scanning technology, it substantially reduces UV-induced photodamage during imaging compared with standard UV fluorescence, which illuminates the full field of view above and below the focal plane. UV two-photon fluorescence images have a high image contrast because the confocal nature of the measurement significantly decreases the background scatter. In addition, this method is compatible with conventional plates and cover slips.

**Figure 7:**
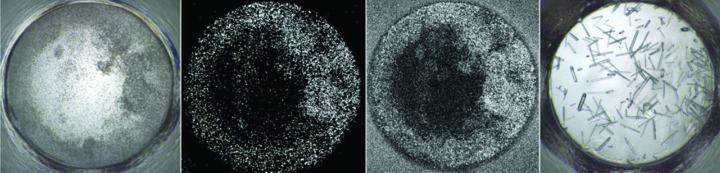
Example of SONICC imaging with two-photon fluorescence. From left to right: visible image of a precipitated drop, SHG image of the same drop showing white signal indicative of chiral crystals, UV-TPEF image of the same drop showing UV fluorescence. The far right image shows optimized crystals produced from optimization of the conditions for the initial nanocrystals identified in the previous images. Each well is 0.9 mm in diameter.

*Implementation in a high-throughput setting*. Both SONICC and two-photon fluorescence have been implemented in a high-throughput manner with routine screening of samples entering the HWI high-throughput crystallization laboratory. In Fig. 7 ▶, an example of the use of these two techniques is shown. Initial visual observation showed one condition that was non-ideal in terms of crystallization outcome; it was precipitated and, while having a granular appearance, was one of many similar results. The SONICC data showed that chiral crystals were present in the visually undefined precipitate and two-photon fluorescence showed that these chiral crystals were protein. Using the initial conditions for the crystallization result, a variant of the drop volume and temperature ratio technique (DVR/T; Luft *et al.*, 2007 ▶) was used and the small crystals were optimized to a volume suitable for in-house or synchrotron diffraction studies.

### 2.2. Other techniques   

The precise three-dimensional position of crystals in a crystallization drop has been studied using Raman spectroscopy by Nitahara *et al.* (2012 ▶). Raman spectroscopy measures shifts in the incident wavelength caused by vibrations, phonons or other excitations in the system under study. However, the technique is time-consuming; this particular study reported needing approximately 1 h to build up an image of about 400 × 200 µm. Nevertheless, the technique was able to clearly image a crystal with 10 µm dimensions. Infrared spectroscopy is extensively used in the study of biological macromolecules. Attenuated total reflection (ATR) Fourier transform infrared (FTIR) spectroscopic imaging (Glassford *et al.*, 2013 ▶) has been used to identify proteinaceous precipitates or crystals (Chan *et al.*, 2009 ▶). As ATR FTIR is a direct-imaging technique, it is faster than Raman spectroscopy and was able to resolve crystals of 40 µm dimensions with the instrumentation used for the study, although the authors note that spatial resolution down to 12 µm is possible. Micro ATR FTIR spectroscopic imaging has been applied to measure crystals grown using the hanging-drop approach (Glassford *et al.*, 2012 ▶). Optical coherence tomography is an interference-based technique that can build up a three-dimensional image of the sample being studied. Nishizawa and coworkers used an ultrahigh-resolution variant of the technique to achieve 2 µm resolution during the observation of protein crystallization experiments (Nishizawa *et al.*, 2012 ▶).

#### 2.2.1. Light scattering   

The Brownian motion of particles in solution is random, but has a velocity distribution dependent on the particle size and concentration, the solution medium and the temperature. This property has been used for light-scattering studies to determine particle size and study the nucleation of protein crystals (Wilson, 2003 ▶). Light scattering can detect the presence (but not the individual locations) of small aggregates (a few nanometres in size; Saridakis *et al.*, 2002 ▶). Dierks and coworkers developed a dynamic light-scattering (DLS) system that could be used on crystallization trays and again showed the growth of aggregates eventually leading to crystals measured directly in the crystallization drop (Dierks *et al.*, 2008 ▶). More recently, Stevenson *et al.* (2014 ▶) used DLS in batch mode (Wyatt DynaPro Plate Reader, ideal for screening large numbers of conditions) to search for the presence of nanocrystals in crystallization drops. To this end, they pipetted 4–6 µl of clear drops, or drops containing granular aggregates, onto DLS plates and were able to detect particles ranging from 50 to 1000 nm for subsequent characterization using TEM. The use of DLS alone does not indicate whether such particles are crystalline in nature. While not a suitable means for locating small crystals at a defined point in space, light scattering is very effective at detecting particles and characterizing their size distribution. It is also an effective technique to probe for potential crystallization conditions and solubility, as reviewed by Wilson & DeLucas (2014 ▶).

### 2.3. Electron microscopy   

Electron microscopy has been used as a technique to study the crystallization process by imaging subunits, defect structure and crystal growth with an effective pixel size of 0.3 nm (Braun *et al.*, 2000 ▶). Transmission electron microscopy (TEM) has been used to study crushed lysozyme crystals, showing crystallites ranging from 0.1 to 0.7 µm in diameter (Gomery *et al.*, 2013*a* ▶); electron diffraction on the same instrument was used to characterize the crystallites in terms of their diffraction properties. Extending this study to scanning electron microscopy (SEM), where the electron beam is focused and scanned over the sample, investigators were able to show the growth process of lysozyme crystals (Gomery *et al.*, 2013*b* ▶). Another study showed that the electron microscope could image growing protein crystals and distinguish them from salt crystals, with the potential to resolve crystals down to 8 nm in size (Maruyama *et al.*, 2012 ▶).

**Figure 8:**
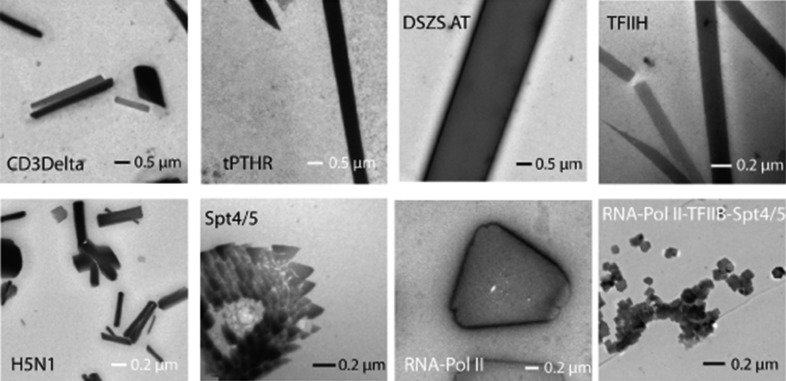
Examples of nanocrystals identified from granular aggregates using negative-stain TEM. For this study, a variety of challenging samples from membrane proteins to multi-protein complexes were used. Samples include three membrane proteins, CD3Delta, thermo-stabilized parathyroid hormone receptor 1 (tPTHR1) and the full-length influenza virus hemagglutinin protein (H5); a soluble protein, *trans*-acting acyl transferase from disorazole synthase (*E. coli*); and four multi-protein complexes, the five-component core of the human transcription factor TFIIH, the yeast heterodimer elongation complex Spt4/5, RNA polymerase II and a complex between RNA-Pol II, the transcription factor TFIIB, Spt4/5 and a 53-mer nucleic acid scaffold. Taken from Stevenson *et al.* (2014 ▶) with permission.

More recently, TEM was used for the specific purpose of enabling nanocrystal detection for XFEL-based studies and to identify nanocrystals from crystallization drops containing granular aggregates (Stevenson *et al.*, 2014 ▶). Both hanging-drop and sitting-drop crystallization experiments were pre-screened using *in situ* UV fluorescence and DLS. Those displaying a positive result were taken directly from the crystallization drop, applied onto a copper grid with continuous carbon film, stained with a 2% solution of uranyl acetate and imaged using an FEI Tecnai T12 transmission electron microscope. In most instances, a single crystallization drop containing thick aggregates was of sufficient concentration for imaging. For the majority of the samples tested (20 in total), TEM visualization (Figs. 8 ▶ and 9 ▶) proved to be an efficient method to reveal whether the samples contained nanocrystals or large protein aggregates. The presence of detergents in crystallization buffers did not appear to have a negative impact on the EM visualization. TEM visualization of crystal lattices allowed the authors to determine whether crystals were protein or salt (including salt crystals coated with protein aggregates that generated false-positive UV signals). Moreover, the use of TEM provided additional insights such as evaluation of diffraction quality through calculation of the Fourier transform of the crystal lattice images. Importantly, this work revealed that submicrometre crystals are ubiquitous in crystallization drops.

In addition to nanocrystal discrimination/visualization, TEM offers the possibility of observing protein behaviour in crystallization drops (Fig. 10 ▶ *a*); this information can be of high value during crystal-optimization experiments (Fig. 10 ▶ *b*). Stevenson *et al.* (2014 ▶) demonstrated the potential of TEM to serve as a fundamental tool for evaluating nanocrystals, as essential as bright-field microscopy is for evaluating and optimizing traditional large crystals.

### 2.4. X-ray analysis in the laboratory   

With the exception of electron microscopy (and the diffraction techniques employed), the techniques discussed above indicate the presence of crystals, their volume and morphology, and whether they consist of macromolecule or salt. Volume and external appearance do not necessarily correlate well with X-ray diffraction properties. Crystals of striking morphology can diffract poorly, while crystals that look ‘ugly’, to use an easily understood term, can diffract extremely well. The highest fidelity imaging method, and ultimately the only relevant method, to detect and characterize crystals is X-ray diffraction.

Before the advent of cryocooling, the standard way to prepare crystals for interrogation with X-rays was to extract the crystal from the medium that it was growing in and place it in a capillary with some suitable liquid to prevent dehydration (Bernal & Crowfoot, 1934 ▶). With counter-diffusion techniques, the crystal is grown inside the capillary, and it has been shown that crystals grown in the capillary can be soaked with an appropriate heavy atom, cryoprotected and used to solve the structure (Gavira *et al.*, 2002 ▶). As home X-ray sources have increased in flux, it has become increasingly possible to study samples *in situ* in sitting-drop crystallization plates, particularly with the development of experimental plates that minimize X-ray absorption. Dedicated laboratory instruments have been produced for this, but have been superseded to some extent by the development of goniometers that can hold and position a whole crystallization plate on a laboratory system (Hargreaves, 2012 ▶) or at the synchrotron (le Maire *et al.*, 2011 ▶).

### 2.5. Crystallization-plate considerations   

The concept of observing crystals as they grow has existed from the early days of macromolecular crystallographic research. Crystallization plates, and the myriad of other containers for crystallization experiments, are optically transparent and designed for visual imaging. With the use of other wavelengths and *in situ* X-ray analysis, the design of appropriate holders for the experiments has necessarily been revisited. In our own laboratory, the 1536-condition screening plates were designed so that the thin film at the bottom of the plate does not interfere with cross-polarized imaging to observe crystal birefringence. Others have designed plates that are made of UV/X-ray transparent film to facilitate not only UV and X-ray screening but also X-ray data collection by eliminating walls around the crystallization drop (Soliman *et al.*, 2011 ▶).

The material from which the plate is made is particularly important for *in situ* studies. In a study of three plates with identical design but formed of different materials (standard polystyrene, the cyclic olefin copolymer Topas 8007 and the cyclic olefin copolymer poly1), significant differences in scattering owing to the materials were seen (Jacquamet *et al.*, 2004 ▶). The polystyrene and Topas 8007 materials had a peak in scattering at ∼5.0 Å, with the standard polystyrene having a peak at ∼4.30 Å but at about 60% of the intensity. In all cases the peak in scattering decayed rapidly as a function of resolution, but the polystyrene displayed noticeable shoulders from 10 to ∼3.3 Å. More recently, plates have improved, with specific designs for *in situ* screening and data collection. These show only a little more background intensity than air (Bingel-Erlenmeyer *et al.*, 2011 ▶). A word of caution: although being able to obtain diffraction from a crystal in a plate is useful, it is also important that the plates used are amenable to dispensing technologies, and to be widely adopted the plates must also be inexpensive, easily stored, stacked and labelled. Other considerations include the rate of evaporation of water or other species through the plate and the quality of the surface on which the crystallization droplet will form.

### 2.6. Summary of observation techniques for crystallization   

Table 1 ▶ summarizes the major observation techniques described above: the name of the technique is given, along with the spatial resolution thus far achieved along with a comparative estimate of the time for the observation and the expense of the instrumentation required.

## 3. Data collection   

### 3.1. Identifying and positioning crystals for data collection   

Identifying crystals and positioning them in the beam is critical for the diffraction experiment. In §[Sec sec2.4]2.4 *in situ* X-ray analysis was discussed, where the whole crystallization plate is placed such that the beam illuminates a crystal or crystals in a single well. This has advantages in that the crystal is maintained within its growth conditions and any physical damage is minimized, but it has disadvantages in that the plate itself may make complete data collection impossible owing to obstruction of the incident (or diffracted) X-rays by the plate, the liquid around the crystal may increase the background and effective signal-to-noise of the data, and such analyses are invariably at room temperature, as the crystal cannot be easily cryoprotected or cryocooled. In most cases, for complete data collection the disadvantages outweigh the advantages and crystals are usually harvested, cryoprotected, cooled and then placed in the beam. The crystal has to be centered within the beam, and the extent of the crystal needs to be defined so that a beam size may be selected to minimize noise from other objects or liquids, *e.g.* a loop or solvent, that may also scatter. Similarly, knowing the size and shape of crystals larger than the X-ray beam size allows helical scanning methods to be used to minimize radiation damage. If injector methods are used, the liquid jet has to be positioned such that it will intersect with the beam. During injector-delivered studies there are considerable advantages to knowing where a crystal is located along the liquid jet to optimize detector readout and even beam delivery.

#### 3.1.1. Optical methods   

Synchrotron beamlines are becoming extremely efficient, with hardware and software designed for remote data collection being developed at many facilities (McPhillips *et al.*, 2002 ▶; Gonzalez *et al.*, 2006 ▶; Soltis *et al.*, 2008 ▶; Beteva *et al.*, 2006 ▶; Warren *et al.*, 2008 ▶; Stepanov *et al.*, 2011 ▶; Fodje *et al.*, 2012 ▶). Essential to this quest for efficiency and ease of operation is the automatic centering of a crystal in the beam. A number of approaches have been adopted from centering of the loop (which assumes that a correctly sized loop was chosen to match the crystal; Pauluhn *et al.*, 2011 ▶) to more sophisticated image-analysis approaches (Pothineni *et al.*, 2006 ▶; Lavault *et al.*, 2006 ▶; Andrey *et al.*, 2004 ▶). These approaches can come close to replicating the manual centering performed by a skilled operator, but may take somewhat longer (Jain & Stojanoff, 2007 ▶).

UV fluorescence has also been used to identify a crystal in the beam. Overexposure to UV light will damage proteins (Fujimori, 1982 ▶) and this damage can be significant enough to be used for phasing purposes (Nanao & Ravelli, 2006 ▶). However, given that the only purpose of putting a crystal on the beamline is for the collection of diffraction data, incidental UV-induced damage needs to be avoided. To overcome this, while still making use of the fluorescent properties of crystals containing tryptophan, Chavas *et al.* (2011 ▶) used low-power UV LEDs and a UV-light detector rather than a camera. They proved that crystals could be detected from the background when they were not obvious by visual imaging.

An imaging system exploiting single-photon excited visible-light emission (Shukla *et al.*, 2004 ▶) has been in use at SSRL BL7-1 since 2012 to aid in visualizing colorless crystals inside loops or on meshes, and has been incorporated into the ‘click-to-center’ video display of the beamline-control software. Protein crystal excitation is in the near-UV and the longer wavelength emission is collected and displayed (Nagarajan, 2014 ▶). Most protein crystals show up brightly on these images, while salt crystals, which are often mistaken for protein crystals when examining video images, remain dark and can be easily avoided (Fig. 11 ▶). It has been observed that protein crystals glow more brightly in these images when dehydrated. This technique does not work well for colored crystals, which often remain dark in the emission images. While nylon 66 and many plastics will also glow, UV-transparent thin-film mounts, *e.g.* those sold by MiTeGen, are essentially invisible.

SONICC microscopy has also been used for centering crystals (Kissick *et al.*, 2013 ▶; Madden *et al.*, 2013 ▶). In the study of Kissick and coworkers, cryopreserved crystals in loops were not visible with optical microscopic observation but were located by X-ray rastering. Transmitted and epi-illumination SONICC and UV-TP imaging was then used on the same loops and the transmitted SONICC signal correlated highly with the X-ray-determined crystal position. Madden *et al.* (2013 ▶) noted that the rastering time per pixel for the nonlinear optics approach gave an ∼10^3^–10^4^ reduction per pixel exposure time compared with X-ray crystal detection.

#### 3.1.2. X-ray-based techniques   

The ultimate measure of the quality of a crystal is not its optical perfection but how well it diffracts. Visual methods can be used to center the loop holding the crystal approximately in the beam path, and low-dose X-rays can then be used to scan over the edge and the profile of the loop to determine and make use of X-ray fluorescence (Karain *et al.*, 2002 ▶) or diffraction itself to find the best position for data collection (Karain *et al.*, 2002 ▶; Song *et al.*, 2007 ▶). This has been extended to a rastering capability incorporated into beamline-control software that allows the user to select an area of the loop and automatically image a discrete grid within the loop using a small beam to identify the best diffracting regions (Cherezov *et al.*, 2009 ▶; Hilgart *et al.*, 2011 ▶). This approach can be rapid, especially with pixel-array detectors, and can be used to detect optically invisible samples or identify multiple samples in, for example, a mesh-like holder (Aishima *et al.*, 2010 ▶). An adaption of the rastering technique is X-ray tomography, which can accurately locate and establish the three-dimensional shape of a crystal in an opaque medium (Warren *et al.*, 2013 ▶). While this used an equivalent X-ray dose to X-ray rastering for a complete 180° study, a more limited set of exposures could provide a suitable result with a significantly reduced dose.

A number of microfocus beamlines offer a raster capability (Flot *et al.*, 2010 ▶; Cherezov *et al.*, 2009 ▶; Hilgart *et al.*, 2011 ▶). For example, at SSRL the highly automated microfocus beamline 12-2 provides a focused beam size of 15 × 50 µm and a minimum collimated micro-beam size of 5 × 5 µm for data collection using small crystals. A low X-ray dose raster-scanning method is incorporated to identify and successfully collect diffraction data from samples with poor optical properties and automatically analyses the resultant diffraction patterns to detect diffraction spots that fit onto a crystal lattice and to score the patterns. The number of these spots detected and the score is overlaid onto the video image of the sample, simplifying crystal identification and alignment into the X-ray beam position (Fig. 12 ▶). When oscillation diffraction images are collected at each raster position, the sample-rotation motor is positioned and accelerated before each X-ray exposure, introducing a large timing overhead. Raster scanning an entire sample loop in this mode can take several minutes to complete depending on the sample size and X-ray beam size. This time can be reduced by collecting a still diffraction image at each raster point using a fast-readout detector, such as the Dectris PILATUS pixel-array detector (PAD), enabling each row of the raster to be collected in a shutterless sweep of the sample (Aishima *et al.*, 2010 ▶).

An X-ray beam can also cause fluorescence analogous to that used for UV-based detection of crystals. Crystals that contain aromatic amino acids emit near-UV fluorescence from delocalized π bonds of the conjugated ring(s) caused by X-ray irradiation. This has been used for crystal centering of a lysozyme crystal in a vitrified solution and also centering from fluorescence caused by a UV source (Gofron & Duke, 2011 ▶).

### 3.2. Monitoring radiation damage   

While optical methods have been used to study crystallization and crystal growth and to center crystals in the X-ray beam, they also have a role in ensuring that the structural data recorded are for the correct biochemical state. X-ray radiation damages biological macromolecules, affecting redox centers, cleaving disulfide bonds and attacking other amino acids. UV–visible microspectrometry can follow some of this damage and determine when the structural data recorded display characteristics associated with radiation damage (Antonyuk & Hough, 2011 ▶; Meharenna *et al.*, 2010 ▶). However, since only about 20% of proteins are colored, Raman difference spectroscopy (RDS) provides a more general, albeit time-consuming, monitoring approach (McGeehan *et al.*, 2011 ▶). Through RDS measurements from the exact portion of the sample being irradiated with X-rays, the reduction of metal sites may be monitored. In the case of an experiment using crystals that are longer than the beam size, the crystal may be translated, exposing a different part of the crystal after an optimal exposure time. When the X-ray beam size is the same or larger than the sample size, one would know if the sample has been compromised and should be replaced.

### 3.3. Summary of centering techniques   

Table 2 ▶ summarizes different detection, positioning or centering techniques suitable for X-ray beamlines. The name of the technique is given along with the spatial resolution thus far achieved and a comparative estimate of the time for the observation and the expense of the instrumentation required.

## 4. Discussion   

Macromolecular crystallization, which underpins X-ray crystallo­graphic structure determination, has been reasonably successful: there are over 100 000 structures in the PDB and the vast majority have arisen from crystals. Most of these studies have come from crystals that were identified and optimized with the aid of visible-light microscopy and (more recently) UV-based techniques. To monitor and study crystallization, a high-quality microscope is still an essential tool. Not only can it be used to identify when crystal hits occur, but a microscope can also be used to classify outcomes into precipitate or skin formation or determine when there is no change at all. Coupled with careful experimental design, this information in itself can be sufficient to identify promising crystallization conditions and suitable directions for optimization. Precipitate may be crystalline; while the addition of a polarizer may help to identify birefringence, it will not distinguish a biological macromolecule crystal from a crystal of salt. Various other methods are available which will distinguish protein from salt crystals that do not require additional instrumentation: the addition of a dye, running a gel on a (dissolved) crystal or simply poking the crystal with a needle to determine whether it is soft (protein) or hard (salt). With the exception of light bulbs, there are no upkeep costs for light microscopy. UV-enabled microscopes are probably the next most accessible option, and are generally sufficient for many structural problems today, but may be subject to specific safety restrictions. Enclosed UV-imaging systems are allowed under most health and safety regulations, but require someone to actually make sure that the imaging happens. Enclosed incubator-based automatic imaging systems provide the most reliable imaging, and can often be equipped with UV or other orthogonal visualization techniques, but have an initial and ongoing cost and will certainly require significant space in the laboratory. Furthermore, these systems also require a data-storage capacity and some (considerable) investment and upkeep of information-technology infrastructure.

**Figure 9:**
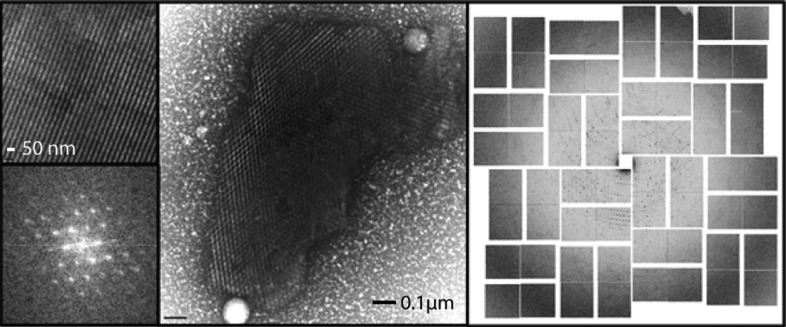
Center, negative-stain TEM image of a complex between RNA polymerase II and green fluorescent protein (GFP). Upper left, crystal lattice; lower left, Bragg spots calculated after applying a Fourier transform showing at least third-order spots. The high lattice quality correlated with diffraction to 4.0 Å resolution at LCLS (right panel). Modified with permission from Stevenson *et al.* (2014 ▶).

**Figure 10:**
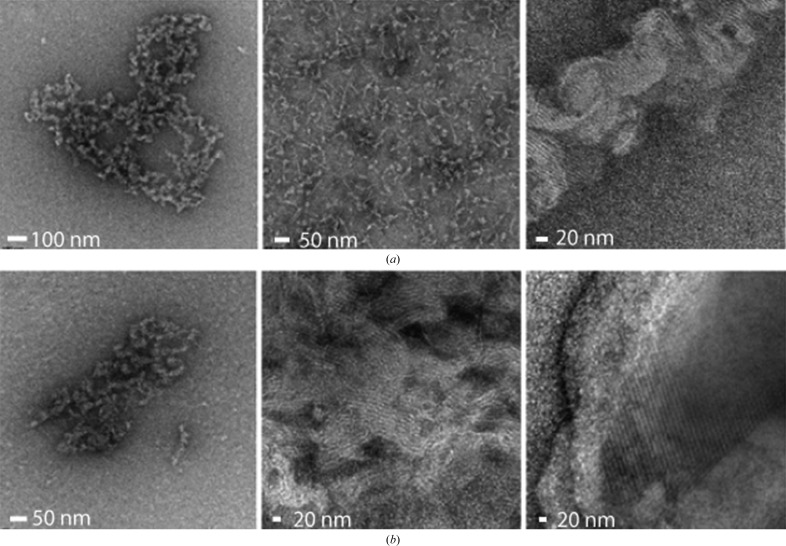
(*a*) Protein aggregates identified in crystallization drops, from disorganized (left panel) and protein streaks (middle panel) to ‘plastic lattices’ (right panel), where evidence of semi-organized lattices can be observed. (*b*) Optimization of crystal conditions for the human PTHR1 receptor can lead from protein aggregates (left panel) and plastic lattices (middle panel) to ordered lattices.

**Figure 11:**
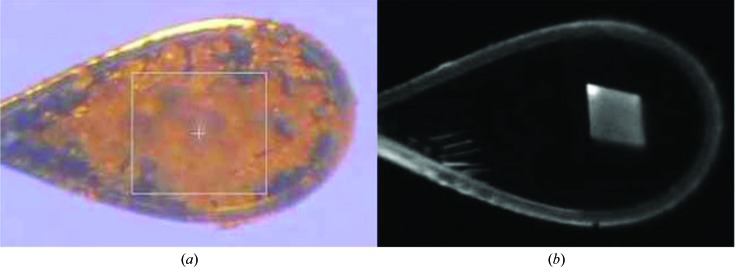
A cryogenically cooled crystal within a nylon loop is obscured by surrounding material when imaged using visible-light microscopy. The white box overlaid on the loop image is 200 × 200 µm in size (*a*). The same protein crystal may be clearly observed using visible-light emission (*b*).

**Figure 12:**
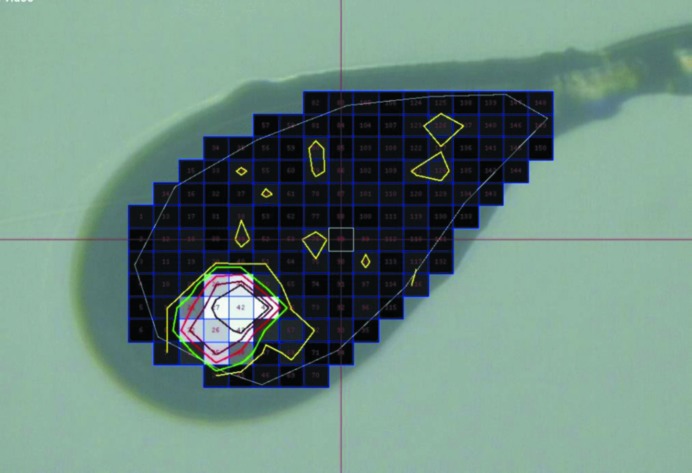
An example of a crystal image with X-ray rastering information overlaid to accurately define the crystal position within the loop. By repeating the process in a perpendicular direction the precise three-dimensional position of the crystal and the strongest diffracting area is obtained. For scale, in this case each rastered pixel is 20 × 20 µm.

With the availability of microfocus beamlines and the advent of X-ray free-electron lasers, opportunities to make use of crystals that challenge visual-light observation techniques are increasing and more sophisticated observation methods are often required. Light scattering, SONICC and EM methods or a combination of these appear to offer reliable solutions when crystals are smaller than can be observed by visible microscopy methods. Light scattering is a diagnostic for the crystallization process in general and is moderately expensive to acquire, but has many applications in the laboratory. It is not beyond what a single laboratory might afford. SONICC and EM are far more expensive in terms of acquisition and upkeep, but provide significantly more information about the crystallization outcome and, in the case of EM, potentially characterize the quality of the crystals. SONICC data are relatively easy to acquire and interpret if an instrument is available. EM experiments showed that it is possible to find multiple nanocrystal conditions in crystallization drops and that there is a good correlation between electron diffraction of negatively stained nanocrystals and X-ray diffraction at LCLS (Fig. 9 ▶). However, limitations to negative-stain techniques using uranyl acetate, including room-temperature diffraction, sample dehydration and possibly crystal cracking owing to interference of the dye with the crystal lattice, restrict electron diffraction to approximately 20 Å resolution in the best of cases. Nanocrystal characterization using cryo-TEM, a well established technique (Nederlof *et al.*, 2013 ▶; Shi *et al.*, 2013 ▶), should allow (i) a more accurate prediction of nanocrystal diffraction, (ii) investigation of whether two or more crystal forms of the same protein diffract at different resolutions and (iii) the establishment of cryo-TEM as a guide for improving nanocrystal quality for XFEL experiments. EM represents the gold standard in imaging crystals; however, it requires both expensive instrumentation and expert staffing. In the near future, it is possible that a dedicated system could be designed that would be more easily affordable than a standard system. Moreover, further development of TEM techniques will allow implementation of high-throughput methodologies using specially designed grids where UV/SONICC-positive crystal drops could be evaluated in an automated fashion.

Given the increasingly sophisticated tools available for crystal-growth setup, detection and testing, it becomes appropriate to think about what is the best way forward for the structural biology community. Realistically, most of the time required to undertake ‘structural biology’ is not spent on X-ray diffraction studies but on producing quantities of a sample that are of sufficient quality for structural studies and performing the biochemical and biological experiments to justify the structural work. Thus, the bulk of the effort should go towards these steps. Is there a need to identify nanocrystals for the structural study to continue? There is evidence to suggest that nanocrystals are sometimes obscured by other outcomes in the crystallization experiment and that their identification can offer a route to optimization for regular synchrotron studies (*e.g.* Fig. 7 ▶). However, it is not yet known whether nanocrystals occur in samples where the conditions cannot be optimized to produce larger crystals, but it is not unreasonable to propose that this may be the case. Both cases offer a justification for identification. There are also specific experimental rationales for the use of nanocrystals, *e.g.* where the sample volume is minimal or for time-resolved studies where a reaction has to occur within the majority if not the whole crystal uniformly. As the sophistication of the imaging technology increases, so does the financial cost; a decision has to be made as to whether these costs are justified by the potential structural knowledge that might be obtained.

Synchrotrons present an interesting model for how sophisticated instrumentation can be provided to the structural biology community by outsourcing expensive (and individually only periodically used) equipment. The concept of outsourcing crystallization is not new; the High Throughput Crystallization Screening (HTS) laboratory at one of our institutes has been setting up and imaging samples in crystallization trials for well over a decade. The benefits of having dedicated platform technologies that can be utilized by many groups are obvious: expensive equipment can be shared, dedicated staff can ensure that the equipment is well maintained and so on. One of the reasons for the success of the HTS screening service is the apparent simplicity of the service: the user has no decisions to make. This model works well for screening and now for high-throughput SONICC imaging, *i.e.* in the HTS laboratory, but becomes problematic for optimization or for any other step that requires thoughtful intervention, steps that are then carried out in the home laboratory. At the C3, the service model has evolved to include both screening and optimization, but with an *à la carte* menu of screens appropriate for initial screening and with optimization options including fine screening, additive screening and seeding, amongst others. UV imaging is available, but SONICC is not. This approach is effective but requires a significant investment of time by the user to make best use of the facility, and this factor alone is a barrier to entry. Given the outlay of investment now needed for instrumentation to identify, locate and characterize crystals that can be used for X-ray data collection, there is a strong argument towards centralized and dedicated facilities.

Over the past decade, automated technologies to mount and position samples on the beamline goniometer, including sample-mounting robots, video-based loop-centering algorithms and X-ray raster searches, have become commonplace at synchrotron sources. More recently, the availability of intense X-ray microbeams and high-speed PAD detectors has further improved sample throughput and the signal-to-noise ratio of the diffraction images collected, enabling identification of and data collection from smaller crystals. However, as the size of the crystals used for data collection is reduced, radiation-damage effects will necessitate the combination of partial data sets from multiple crystals, bringing about new challenges. In particular, problems with crystal non-isomorphism can make it difficult to produce useful data sets by combining data from multiple crystals, and methods optimized to produce, identify and deliver groups of well diffracting isomorphous crystals to the synchrotron or XFEL X-ray beam are important areas for future development.

Our motive for this article has been the stunning decrease in the volume of crystal samples needed to obtain diffraction data and the realization that visible microscopy can no longer resolve samples that might provide useful data at an XFEL source. While these sources are producing scientific breakthroughs, they are few and far between, only one or a few planned experiments can be run at any time, and the efficiency of their operation with respect to synchrotron studies is poor. Based on this, the need for observation techniques beyond the visible microscope may seem to be a low priority in terms of characterizing crystals for data collection; however, it is likely that as more XFELs come online and these are joined by upgraded microfocus synchrotron beamlines there will be a substantial increase in the availability of facilities capable of collecting useful data from crystals of a few micrometres or smaller in size. A more pressing application is in enabling studies of samples that otherwise would not have gone on to be used for X-ray diffraction. Fig. 7 ▶ illustrates this, where the initial visible image did not look promising. Once it was known that crystals were present, optimization produced diffraction-quality crystals. As sources able to make use of nanocrystals increase and the facilities necessary to detect them become more common, single-crystal work at synchrotrons will also benefit from visualization techniques that detect success beyond the capabilities of visible microscopes.

## 5. Conclusion   

X-ray crystallography is rapidly evolving and it is a statement of this evolution that we are now able to make use of crystals that are smaller than the wavelength of light. This requires the evolution of laboratory techniques to extend beyond the optical microscope and to make use of many complementary techniques. In a manner akin to how the synchrotron has largely replaced the laboratory X-ray source for structural studies, so will techniques that extend the observation of crystals beyond the optical microscope enhance structural biology in the future.

## Figures and Tables

**Table 1:** Summary of the major observation techniques for identifying crystals and following the crystallization process

Technique	Spatial resolution achieved	Time	Expense	Outcome
Eye	∼500 µm	Rapid	None	Identification of the presence and position of large crystals. Measurement of number and size.
Visual microscopy	∼2 µm	Rapid	Low	Identification of the presence and position of large and small crystals. Measurement of number and size
UV microscopy	∼2 µm	Medium	Medium	Identification of the presence and position of large and small crystals. Measurement of number and size. Distinguishes between biological and salt crystals in many cases.
Light scattering	A few nanometres	Medium	Medium	Characterization of size distribution. No number or positional information.
SONICC	∼0.2 µm	Medium	High	Identification of the presence and position of large to nanocrystals. Measurement of number and size. With TP-UV, distinguishes between biological and salt crystals
Electron microscopy	50–100 Å, (individual proteins) ∼5–10 µm	Long	Very high	Identification of the presence and position of large to nanocrystals. Measurement of number and size. Distinguishes between macromolecular and salt crystals. Characterization of diffraction quality.

**Table 2:** Summary of the major detection, positioning or centring techniques on the beamline

Technique	Spatial resolution achieved	Time	Expense	Outcome
Optical microscopy	∼2 µm	Rapid	Medium	For visible crystals the crystal can be centered accurately.
UV fluorescence	∼2 µm	Rapid	Medium	If the crystal fluoresces it can be centered accurately.
Visible two-photon fluorescence	∼2 µm			Similar to UV.
SONICC	∼2 µm	Medium	Very high	Similar to UV.
X-ray rastering	Sensitive to the diffraction properties despite the size (depends on the smallest available X-ray beam size)	Short	High	Not only can the crystal be centered but the spatial diffraction properties can be characterized.
